# Harnessing dendritic cells for pancreatic cancer immunotherapy: a novel promising approach

**DOI:** 10.1002/mco2.70066

**Published:** 2025-01-14

**Authors:** Hui Zeng, Yidong Wu, Xinghua Long

**Affiliations:** ^1^ Department of Laboratory Medicine Zhongnan Hospital of Wuhan University Wuhan China; ^2^ Center of Clinical Laboratory Hangzhou Ninth People's Hospital Hangzhou China

1

In a recently published study in Science, Mahadevan et al. offer a promising breakthrough in cancer immunotherapy by elucidating the pivotal role of Type I conventional dendritic cells (cDC1s) in enhancing immune checkpoint blockade therapy (iCBT) in pancreatic ductal adenocarcinoma (PDAC).^1^

PDAC remains a formidable challenge with a dismal prognosis. Immunotherapy, which harnesses the body's own immune system to fight cancer, holds immense promise. However, several aspects of PDAC limit the efficacy of immunotherapy^2^, ^3^:

1. The immunosuppressive microenvironment: the pancreatic cancer tumor microenvironment exhibits strong immunosuppressive characteristics. Tumor cells promote the activation of surrounding stromal cells and immunosuppressive cells, including regulatory T cells (Tregs), myeloid‐derived suppressor cells (MDSCs), and tumor‐associated macrophages (TAMs).
2. Low tumor mutational burden (TMB):Neoantigens play a crucial role in facilitating lymphocyte infiltration and augmenting the sensitivity of iCBT. Nevertheless pancreatic cancer is characterised by a relatively low TMB. This paucity of somatic mutations restricts the generation of neoantigens, subsequently diminishing the sensitivity of immunotherapy.
3. Antigen presentation disorder: Most DCs in PDAC exhibit an immature phenotype accompanied by feeble viability. These DCs are inept at efficiently presenting tumor antigens to effector T cells, thereby impairing antigen recognition and subsequent T cell activation.

DCs, as key orchestrators of immunity and tolerance, play a crucial role in cancer immunotherapy by presenting antigens to T cells and secreting cytokines. DCs comprise diverse subsets, each with distinct functions and antigen‐presenting abilities. The migration of DCs to lymph nodes is crucial for T cell‐antigen interaction. Specifically, cDC1s demonstrate superior migratory ability,^4^ excelling in cross‐presentation, which firmly establishes them as vital antigen‐presenting cells. cDC1s capture antigens from deceased tumor cells, transport them to draining lymph nodes, and present them to CD8+ T cells, initiating an anti‐cancer immune response (Figure 1A). This process is considered foundational for inducing anti‐cancer CD8+ T cells.

**FIGURE 1:**
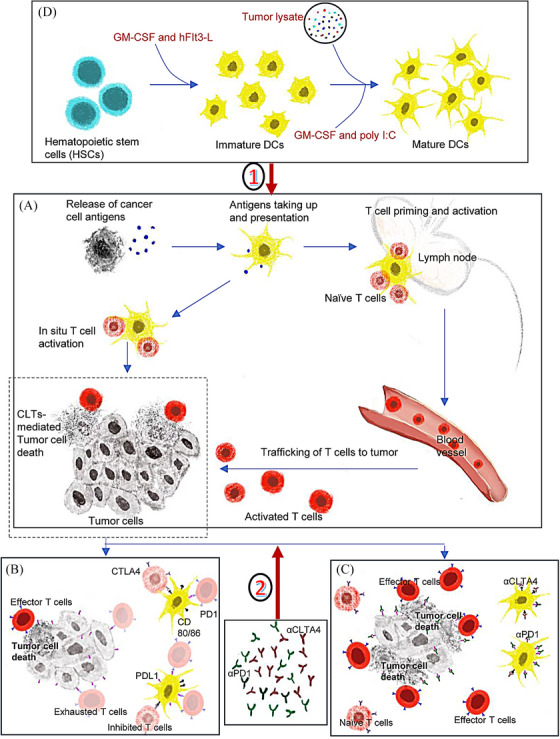
The mechanisms of type I conventional dendritic cells (cDC1s) vaccine combined with immune checkpoint blockade therapy (iCBT). (A). cDC1s mediated anti‐tumor immunity. cDC1s present tumor antigens to T cells in lymph nodes and also interact with T cells in situ. (B). Programmed death‐ligand 1‐Programmed death‐1 (PDL1‐PD1) and CD80‐cytotoxic T‐lymphocyte associated protein 4 (CD80‐CLTA4) mediated T cell impairment. (C). Enhanced anti‐tumor activity. (D). cDC1s vaccine preparation. Incubating bone marrow cells in cRPMI (10% heat‐inactivated fetal bovine serum (FBS), 1% penicillin‐streptomycin (PS), 1 mM sodium pyruvate, and 50 mM b‐mercaptoethanol) supplemented with GM‐CSF combined with hFIt3‐L for 9 days and subsequently replating with the same combination of cytokines and harvesting at day 15–17. The harvested cells are then used for co‐stimulation with tumor lysate in media supplemented with poly I:C and GM‐CSF for 4 hours. 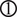: cDC vaccine enhances the magnitude of antigen presentation and enlarges the frequency of effector CD8+ T cells. 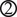: iCBT blocks the inhibitory signals mediated by the checkpoints and enhances the trafficking and retention of effector T cells.

Moreover, cDC1s are rich in T‐cell co‐stimulatory factors.^5^ They modulate immune cell migration and recruit T cells in situ through various chemokine‐receptor interactions. Additionally, cDC1s secrete cytokines that support the tumor immune microenvironment. Combining cDC1s with iCBT enhances anti‐tumor responses in multiple ways. For example, cDC1s enhance anti‐PD‐1 therapy in an IL‐12‐dependent manner.^5^ Crosstalk between cDC1‐secreted cytokines and those produced by CD8+ T cells is crucial for reactivating exhausted T cells and subsequent anti‐tumor responses.^5^

Mahadevan et al. discover that during pancreatitis, cDC1s frequency increases while T cells are suppressed. Activated cDC1s present self‐antigens, suppressing CD4^+^ and CD8^+^ T cell infiltration and mitigating autoimmune tissue destruction. In PDAC associated with pancreatitis (ptPDAC), activated cDC1s frequency increases without T cell suppression. Spatial proximity between cDC1s and CD4^+^ T cells increases, with most infiltrating CD4^+^ T cells being Tregs, TH2, and TH17 subsets. Activated DCs triggered a tolerogenic CD4+ T cell reaction, promoting tumor initiation. However, specific depletion of CD4^+^ T cells elicits a potent CD8^+^ cytotoxic T cell response and inhibits tumorigenesis. Inflammation in ptPDAC accelerates cancer progression but provides immune traits favorable for immunotherapy. A cDC1 vaccine loaded with PDAC antigens enhances iCBT efficacy, induces functional CD8+ T cell memory responses to prevent recurrence, and offers a target for improving PDAC treatment.

The study highlights the mechanisms through which cDC1s exert their effects including antigen presentation and cytotoxic T cell activation key processes for targeting and destroying cancer cells.^1^ Enhancing cDC1 function or numbers in tumors could improve immunotherapeutic outcomes. A cDC1 vaccine broadens tumor antigen diversity, enhances antigen‐presenting, and reshapes immune infiltration by recruiting more oligoclonal T‐cell populations to target a wider range of tumor antigens. Adding iCBT removes PD‐1/CLTA‐4‐mediated suppression on T cells, boosting tumor‐specific clone trafficking and retention at the tumor site (Figure 1B,C).

While promising, this research opens several avenues for future investigation. Understanding the precise signals and pathways that recruit and activate cDC1s in the tumor microenvironment will be crucial. Additionally, there are some challenges to translating these findings into clinical practice:

1. Acquisition (Figure 1D) and evaluation of cDC1 vaccine: Preparing vaccines at scale while ensuring proper differentiation and maintaining phenotype/function is challenging. Vaccine evaluation and measurement pose an additional obstacle. DCs plasticity and heterogeneity complicate the precise classification. Standardized evaluation metrics are lacking.
2. Antigen loading: Selecting an appropriate antigen‐loading method is of crucial significance. Using patient tumor lysates allows personalized individualized treatment but hikes costs. The lysates` antigenic mixture, including tumor‐specific, ‐associated, and normal tissue antigens, leads to an unpredictable immune response. The immune system may have mechanisms in place to prevent an autoimmune response against self‐antigens, which can undermine the ability of the vaccine to mount an effective anti‐tumor response.
3. Vaccine delivery: Determining the optimal route (intravenous vs. intratumoral) and depth requires careful consideration of immune responses and potential toxicity.
4. Possible toxic side effects: Combining the cDC1 vaccine and iCBT risks overactivation of the immune system, potentially leading to autoimmunity or inflammation‐related toxicity.
5. Regularities of drug use related to interval and dose: Optimal dosing intervals, dosage, sequences, and combinations with iCBT remain unclear.
6. Challenges related to patient selection: Tumor immunology heterogeneity complicates treatment. Combination therapy may benefit patients with specific immunophenotypes but poses challenges for advanced cases with a low count of tumor‐infiltrating lymphocytes, high level of immunosuppressive cell infiltration, and low TMB.

The research conducted by Mahadevan et al. marks substantial progress in understanding PDAC immunotherapy. By emphasizing the role of cDC1s, the study enriches the understanding of tumor immunology and lays the groundwork for novel therapeutic strategies. However, further investigations are essential to ensure the safety and efficacy of these strategies in clinical trials. Deciphering the mechanisms underlying cDC1s and iCBT‐mediated immunity will be critical for maximizing clinical benefits.

## AUTHOR CONTRIBUTIONS

H.Z. drafted the manuscript, Y.W. drew the figure, and X.L. drafted and reviewed the manuscript. All the authors read and approved the final manuscript.

## CONFLICT OF INTEREST STATEMENT

The authors declare no conflict of interest.

## ETHICS STATEMENT

Not applicable.

## Data Availability

Not applicable.

## References

[1] Mahadevan KK , Dyevoich AM , Chen Y , et al. Type I conventional dendritic cells facilitate immunotherapy in pancreatic cancer. Science. 2024;384(6703):eadh4567. 38935717 10.1126/science.adh4567 PMC11841451

[2] Balachandran VP , Beatty GL , Dougan SK . Broadening the impact of immunotherapy to pancreatic cancer: challenges and opportunities. Gastroenterology. 2019;156(7):2056‐2072. 30660727 10.1053/j.gastro.2018.12.038 PMC6486864

[3] Bear AS , Vonderheide RH , O'Hara MH . Challenges and opportunities for pancreatic cancer immunotherapy. Cancer Cell. 2020;38(6):788‐802. 32946773 10.1016/j.ccell.2020.08.004 PMC7738380

[4] De Vries IJ , Krooshoop DJ , Scharenborg NM , et al. Effective migration of antigen‐pulsed dendritic cells to lymph nodes in melanoma patients is determined by their maturation state. Cancer Res. 2003;63(1):12‐17. 12517769

[5] Garris CS , Arlauckas SP , Kohler RH , et al. Successful anti‐PD‐1 cancer immunotherapy requires T cell‐dendritic cell crosstalk involving the cytokines IFN‐gamma and IL‐12. Immunity. 2022;55(9):1749. 36103861 10.1016/j.immuni.2022.07.021

